# Family’s Caregiving Status and Post-Stroke Functional Recovery During Subacute Period from Discharge to Home: A Retrospective Study

**DOI:** 10.3390/jcm13226923

**Published:** 2024-11-17

**Authors:** Jungmin So, Moon-Ho Park

**Affiliations:** Department of Neurology, Korea University Ansan Hospital, Ansan 15355, Republic of Korea; wjdalsth1029@gmail.com

**Keywords:** family caregiving, stroke, recovery, home, subacute

## Abstract

**Background/Objectives**: Family members, often informal caregivers, play a crucial role in providing home care for stroke survivors. The period following discharge to home after receiving acute stroke management in a hospital includes the subacute phase of stroke and remains critical to the recovery of stroke patients. This study evaluated the association between family caregiving and post-stroke functional recovery after discharge to home. **Methods**: Data from 402 patients with stroke were obtained from the Korea University Ansan Hospital Stroke Center from January 2019 to May 2022. The family’s caregiving status was evaluated for family burden and supportable environment in the home. This study’s outcome of interest was the modified Rankin Scale (mRS) at discharge to home and three months after stroke onset. The repeated mRS scores were analyzed using the Linear Mixed Model. **Results**: Median days from discharge to 3 months after stroke onset was 81.0 days. The median score of mRS at discharge was 2.0, and the score at three months after stroke onset was 2.0. The distribution of mRS score 0–2 was 60.9% at discharge and 72.1% at three months after stroke onset. In Linear Mixed Models after adjustments with covariables, the family’s caregiving status was positively associated with repeated mRS scores (β = 0.17; 95% confidence interval = 0.11, 0.23; *p* < 0.001). **Conclusions**: These findings suggest that family caregiving to patients with stroke might be associated with post-stroke functional recovery within the period from discharge to home until three months after stroke onset.

## 1. Introduction

Stroke is the second most common cause of death [1] and the third most common cause of disability worldwide [2]. The global prevalence of stroke was 33 million [3] and, the overall incidence of stroke was 232 subjects per 100,000 in South Korea [4]. Although many efforts have been made to improve the management of stroke, it still has a high risk of unfavorable prognosis, and most stroke survivors have long-term functional deficits [1]. After acute stroke treatment in a hospital, more than three-quarters of patients with stroke are eventually discharged to home [5]. However, most of those who return home still require continuous rehabilitation and assistance with activities of daily life. Considering sociodemographic status or sometimes insufficient social welfare services, many patients with stroke are provided such care by family members who function as informal caregivers. In Asia, most caregivers are the patients’ parents, spouses, children, or other family members [6,7].

The period following discharge to home after acute stroke onset, including the subacute phase (two to three months after stroke), remains a dynamic period and is also critical for the recovery of stroke patients [8,9]. The role and support provided by family members at home could become crucial factors for stroke patients.

Numerous previous studies on family members of stroke patients have emphasized family members’ quality of life or caregiver burden, neuropsychiatric aspects such as anxiety or depression, or the nature and economic costs of family care provided to stroke patients [10,11,12]. Family caregiving to patients, which has been relatively overlooked, can be another important factor for patients with stroke [13,14].

The objective of this study was to evaluate the association between the caregiving status of stroke patient family and the prognosis of the subacute phase after discharge to home. We hypothesized that a supportive family caregiver status would show favorable post-stroke functional recovery during the subacute period from discharge to home.

## 2. Materials and Methods

### 2.1. Study Design

This was a retrospective study comparing family caregiving status and post-stroke functional recovery at discharge and at 3 months after stroke onset among patients who had been discharged to home after hospitalization. In this study, family caregiver refers to an unpaid family member, spouse, or child who assumes the primary responsibility of caring for a stroke survivor in-house and who lives with the stroke survivor after hospital discharge. The post-stroke functional recovery was assessed by the change in the modified Rankin Scale (mRS) which is the most commonly used measure of recovery [15,16]. We investigated the effects of family caregiving status on the longitudinal change of post-stroke functional recovery over 3 months after stroke onset.

### 2.2. Inclusion and Exclusion Criteria 

The participant inclusion criteria were as follows: (1) patients were diagnosed with ischemic stroke and confirmed by a brain MRI corresponding to stroke symptoms and (2) stroke patients were discharged to home after hospitalization. The exclusion criteria were as follows: (1) patients with hemorrhagic stroke, (2) incomplete, refusal, or omission of tests, (3) transient ischemic attack, (4) hospital days ≥ 3 months, and (5) failure to complete follow-up.

### 2.3. Data Collection

From a prospective institutional database (Korea University Stroke Registry) [17], we retrospectively selected consecutive patients with stroke who were admitted to the stroke center of the Korea University Ansan Hospital between January 2019 and May 2022. We obtained data from the stroke patients who were discharged to home for research purposes in an anonymized form. For the analyses, we collected data in three main parts:

(1) Information on demographics: Age, sex, education, body mass index (BMI), and other data were collected through the electronic database system.
(2) Covariate data: The following clinical data were collected for each patient, including routine laboratory tests, brain imaging, and stroke assessment. Hypertension was defined as a systolic blood pressure of at least 140 mmHg, diastolic blood pressure of at least 90 mmHg, or as having received a previous diagnosis or prescription for antihypertensive medication. Diabetes mellitus was defined as a fasting serum glucose level ≥ 126 mg/dL, a non-fasting serum glucose level ≥ 200 mg/dL, a hemoglobin A1c level ≥ 6.5%, or a history of insulin therapy and/or oral hypoglycemic drugs. Atrial fibrillation was defined as persistent atrial arrhythmia, with irregular R-R intervals and no clear repetitive P waves, and was diagnosed with an electrocardiogram, 24 h Holter, or continuous electrocardiogram monitoring during hospitalization. Dyslipidemia was defined as having at least one of the following conditions: low-density lipoprotein cholesterol ≥ 160, the use of a lipid-lowering drug, triglycerides ≥ 200, or high-density lipoprotein cholesterol < 40 mg/dL.

The type of ischemic stroke was classified according to the Trial of Org 10,172 in the Acute Stroke Treatment (TOAST) classification system [18]. The subtypes were classified into five categories based on etiology using the TOAST classification: large artery disease, cardioembolism, small vessel occlusion, stroke of other determined etiology, and stroke of undetermined etiology. The undetermined etiology category included three heterogeneous groups: two or more causes, negative evaluation, and incomplete evaluation. The extent of the diagnostic workup and stroke subtypes were determined primarily by the stroke neurologists in charge of the patients. Stroke subtypes were confirmed at a monthly stroke registry meeting.

Some clinical features that were potentially related to vascular diseases were categorized as abnormal levels using the following criteria: anemia was defined as hemoglobin <12 g/dL in females and <13 g/dL in males; reduced kidney function as decreased estimated glomerular filtration rate (eGFR) according to the Chronic Kidney Disease Epidemiology Collaboration formula (eGFR < 60 mL/min per 1.73 m^2^) [19]; and advanced white matter changes were defined using a proposed visual rating scale [20].

The pre- and post-stroke documented use of anti-thrombotic medications such as anti-platelets (aspirin, clopidogrel, cilostazol, triflusal, or their combinations) and oral anticoagulants (warfarin, dabigatran, apixaban, rivaroxaban, or edoxaban) were collected.

The Montreal Cognitive Assessment (MoCA) [21] was used for neuropsychological evaluations. Higher MoCA scores represent better performance on neuropsychological tests.

(3) Research data: This segment collected patient conditions, family caregiving status, and functional recovery following discharge. The family’s caregiving status was assessed by the following four questions: (1) “The family caregiver must care for the patient for at least 8 h a day”. (2) “The patient cannot go out without the help of a family caregiver”. (3) “The patient must stay home alone without a family caregiver”. (4) “The family caregiver also has difficulty moving due to their illness”. These questions were modified from the part of the physician’s referral for the Long-term Care Insurance Act in Korea [22,23]. The answer categories were recorded using a 3-point Likert-type scale ranging from 0 to 2 (0 = none, 1 = 1–2 times a week, 2 = more than 3 times a week). Based on these four questions, the final family caregiving status will be summarized as between 0 and 8, with higher scores representing worsened family burden and worsened supportable environment in the home.

The post-stroke functional recovery was assessed by the change in mRS which measured functional outcomes using a seven-point Likert scale, from 0 (no symptoms) to 5 (severe disability) and 6 (dead) [15,16]. The mRS at discharge (discharge mRS) and the mRS at three months (90 days) after stroke onset (3-month mRS) were assessed. The longitudinal mRS score was set as the difference between two mRS scores: 3-month mRS score–discharge mRS score. Less than zero of difference represents favorable post-stroke functional recovery.

### 2.4. Statistical Analysis

Continuous variables were presented as medians and interquartile ranges (IQR), and categorical variables were frequencies and percentages, as appropriate.

To investigate the effects of family caregiving status on repeated mRS scores, we performed univariate and multivariate Linear Mixed Models with a first-order autoregressive (AR1) covariance structure, including a random intercept for each patient. The scores of mRS at discharge and at 3 months after stroke onset were used as the dependent variables. Random effects for intercepts and slopes were included. The model was adjusted for confounding factors, such as demographic and clinical data, which were selected in the initial univariate analysis with cut-off *p* values of 0.05. The multivariate models included fixed effects of time (measured in days from discharge) as well as family caregiving status and its interactions with time.

Statistical significance was declared when the two-tailed *p* value was <0.05. SPSS (version 20.0; SPSS for Windows, IBM Corp., Armonk, NY, USA) and R (version 4.3.3; R Foundation for Statistical Computing, Vienna, Austria) were used for all statistical analyses.

## 3. Results

### 3.1. Patient Characteristics

**Figure 1:**
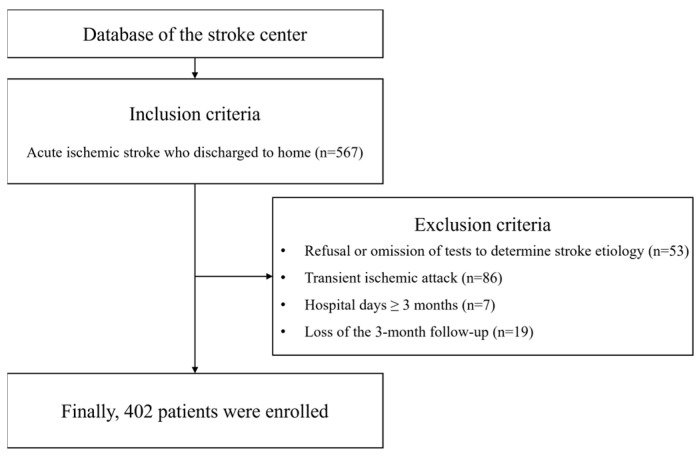
Flow chart describing study participant selection.

A total of 567 patients with stroke were enrolled, and 165 were excluded from the final analysis based on prespecified criteria as reported in the flow chart (Figure 1).

The final analysis included 402 patients. The median age was 71.0 years, and almost 61.4% were males. The median number of days from discharge to 3 months after stroke onset was 81.0 days. Regarding stroke characteristics, 25.1% presented with large artery disease, 20.1% with cardioembolism, and 24.9% with small vessel occlusion. Clinical characteristics are shown in Table 1.

### 3.2. mRS Scores at Discharge and Three Months After Stroke Onset

**Figure 2:**
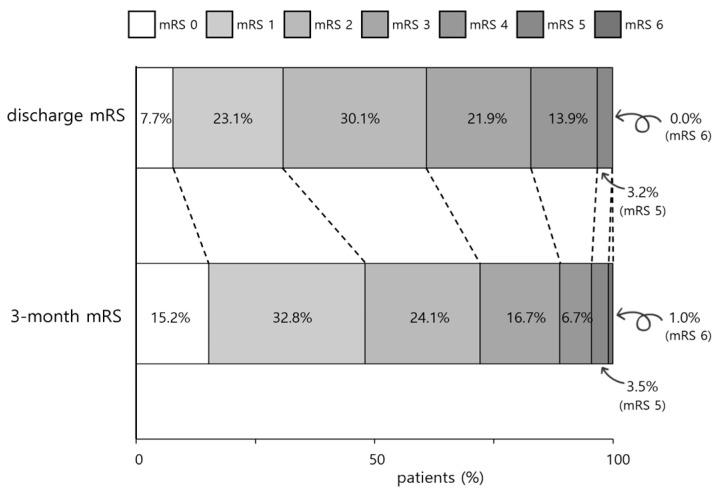
Distribution of mRS scores at discharge and three months after stroke onset.

The detailed distribution of mRS scores at discharge and three months after stroke onset is shown in Figure 2. The median [IQR] score of mRS at discharge was 2.0 [1.0–3.0], and the score at three months after stroke onset was 2.0 [1.0–3.0]. The distribution of mRS score 0–2 was 60.9% at discharge and 72.1% at three months after stroke onset.

### 3.3. Longitudinal Analysis

The univariate Linear Mixed Model showed a direct association between repeated mRS scores and family caregiving status [β = 0.24; 95% confidence interval (95%CI) = 0.19, 0.29; *p* < 0.001]. Among demographic and clinical data, age (β = 0.02; 95%CI = 0.01, 0.03; *p* = 0.001), small vessel occlusion (β = −0.45; 95%CI = −0.79, −0.11; *p* = 0.010), atrial fibrillation (β = −0.34; 95%CI = −0.64, −0.04; *p* = 0.025), anemia (β = −0.30; 95%CI = −0.58, −0.02; *p* = 0.037), white matter changes (β = −0.43; 95%CI = −0.75, −0.12; *p* = 0.008), IV thrombolysis (β = −0.63; 95%CI = −1.03, −0.22; *p* = 0.003), IA thrombectomy (β = −0.76; 95%CI = −1.47, −0.06; *p* = 0.033), and MoCA (β = −0.05, 95%CI = −0.07, −0.04, *p* < 0.001) were found as potential confounding factors.

The multivariate Linear Mixed Model, adjusted for potential confounding factors, confirmed the association between repeated mRS scores and family caregiving status (β = 0.17; 95%CI = 0.11, 0.23; SE = 0.029; t = 5.75; *p* < 0.001) (Table 2). No interaction was observed between family caregiving status and time from discharge to 3 months after stroke onset.

## 4. Discussion

This study reveals that the family’s caregiving status was found to be significantly associated with post-stroke functional recovery during the subacute period from discharge to home until three months after stroke onset. The findings of this study indicate that better family caregiver status tends to be favorable for post-stroke functional recovery at home.

Post-stroke functional recovery is a complex, dynamic, and multifactorial process in which an interplay of genetic, pathophysiologic, sociodemographic, and therapeutic factors determines the overall recovery trajectory [8,9]. This study introduces the family’s caregiving status as an associated factor in post-stroke functional recovery during the subacute period, which was up to three months after stroke onset. The subacute period is also shown to be a crucial period for post-stroke functional recovery, as was the acute period which was less than 30 days after stroke onset [8,9].

Following acute stroke management in a hospital, most patients with stroke are discharged to homes in the community [5]. These patients require ongoing rehabilitation management or assistance for activities of daily living, but many might find it difficult to sustain this management themselves because they face patient factors such as decreased independence or mobility, low self-esteem, loss of social relationships, and social factors such as insufficient social welfare services or traditional attitudes such as racial-ethnic factors in non-Western or developing countries [24,25]. Therefore, after receiving acute stroke management in a hospital and discharge to home, family members should support the primary caregiver through direct or indirect assistance in rehabilitation and caregiving during the subacute and chronic periods as well as manage tasks including bathing, dressing, and taking medicine [25]. Moreover, public health policies have increasingly emphasized community-dwelling models such as long-term care insurance systems or social care so that patients can continue to live in their own homes longer instead of placing them in institutions [26]. Thus, the family plays an important role in accessing health services in the home as well as in local communities.

Regarding family and stroke prognosis, most prior studies evaluated various psychological aspects including depression, sleep disturbance, loneliness, stress, or family burden or well-being related to providing care [10,11,12]. Prior studies have reported that social family support or home-based support, which does not involve direct family members providing caregiving but indirect social service to family members or home-based supportive interventions to build family support using health education to increase physical activity and emotional support, love, warmth, and family relationships, improved psychological outcomes for both patients and caregivers [27,28,29,30]. Yet, there have been conflicting findings, with some studies finding no positive association between these support activities and functional outcomes [27,28] and others revealing beneficial effects of supportive interventions and functional outcomes [29,30]. Some prior studies also reported that a home care model that provided guidance and training for patients and their family caregivers at home was associated with reduced dependency among patients with stroke in China [31], and home healthcare services have proven effective in fulfilling the needs of disabled elderly in Japan [32].

Recently, it was reported that stroke prognosis did not reveal differences between patients cared for by family members and those cared for by professionals [33]. Regarding direct family caregiving to patients with stroke and patients’ prognosis, the results of this study concurred with others that better family caregiving status was associated with improved functional outcomes for patients with stroke [25,34]. Moreover, a family-based program for post-stroke patients and their families showed significant improvements in post-stroke functional recovery [25]. If family caregivers can learn to acquire caregiving skills and rehabilitation techniques during hospitalization, they are better positioned to assist stroke patients at home [33].

Post-stroke functional recovery is optimized if the family’s caregiving status is better such as a healthy and supportive family [35]. The supportive family is an important factor in enabling stroke patients to remain in the community [36]. One study reported that a family’s caregiving, as well as low income and lack of insurance, was associated with a poorer prognosis of stroke [37]. However, that study simply compared family caregiving and healthcare assistants without considering family status, so the family caregiving group may have likely had lower socioeconomic status or less medical knowledge. Therefore, it is difficult to directly compare the results of the present study which evaluated family status including family burden and support environment.

This study has certain limitations. The first is that this was a single-center, retrospective study, and external validation of the results may be needed. Potential problems with the retrospective approach include selection bias and misclassification bias because of the retrospective nature of the study. Therefore, future multicenter studies with larger cohorts should be conducted. Second, regarding the family’s caregiving status, this scale should verify psychometric properties such as reliability and validity. Third, the concept and roles of family can vary according to socio-cultural factors. Ranging from simple cohabitants to comprehensive relationships, this diversity is due to socio-cultural differences. These factors and their roles in stroke outcomes are an understudied area of stroke research and warrant further research. Fourth, this study performed evaluation during the subacute period. A longitudinal study is needed to determine whether family caregiving continues to be associated with functional recovery after the subacute period. Fifth, in common with other retrospective studies, the a priori power calculation of the sample size was not carried out, and the strategy was just to collect the maximal number of informative cases. Sixth, this study did not evaluate various medications for primary and secondary stroke prevention, such as anti-hypertensive, diabetes, or statins nor the dosage and frequency of pre- and post-stroke anti-thrombotics. Seventh, although the mRS was the most commonly used measure of recovery, the measurement of post-stroke functional recovery is complex because the definition of recovery is highly variable across measures, and trying to approach it from a purely quantitative angle does not adequately address this complexity.

## 5. Conclusions

This study revealed that the family’s caregiving status could be associated with post-stroke functional recovery during the period from discharge to home until three months after stroke onset. The family’s caregiving, unlike well-established biological risk factors, should be carefully assessed as a potential factor because it can be influenced by socio-cultural diversities. Therefore, significant further research in this area of stroke recovery could optimize post-stroke outcomes. It is important to note that understanding the factors influencing post-stroke functional recovery can be incorporated into multifactorial and individualized approach plans for patients with stroke. These promising findings should be verified and expanded through large randomized control trials that compare family caregiving to routine post-discharge care.

## Figures and Tables

**Table 1:** Demographic and clinical characteristics.

Characteristics	(*n* = 402)
Age, years	71.0 [60.0–79.0]
Sex, men	247 (61.4%)
Education, years	
Low (≤6)	189 (47.0%)
Intermediate (6–12)	173 (43.%)
High (≥12)	40 (10.%)
BMI, kg/m^2^	
Underweight (<18.5)	19 (4.7%)
Normal (18.5–22.9)	149 (37.1%)
Overweight (23.0–24.9)	95 (23.6%)
Obese (≥25.0)	139 (34.6%)
Current smoking	96 (23.9%)
Days from discharge to 3 months after stroke onset	81.0 [77.0–84.0]
TOAST	
Large artery disease	101 (25.1%)
Cardioembolism	81 (20.1%)
Small vessel disease	100 (24.9%)
Stroke of other determined etiology	26 (6.5%)
Stroke of undetermined etiology	94 (23.4%)
Hypertension	266 (66.2%)
Diabetes mellitus	139 (34.6%)
Atrial fibrillation	80 (19.9%)
Dyslipidemia	202 (50.2%)
Pre-stroke medication	
Anti-platelets	118 (29.4%)
Anticoagulants	13 (3.2%)
Anemia	93 (23.1%)
WBC, ×10^3^/μL	7.50 [6.11–9.35]
Reduced kidney function (eGFR < 60 mL/min per 1.73 m^2^)	64 (15.9%)
White matter changes	67 (16.7%)
Recanalization	
Thrombolysis, IV	38 (9.5%)
Thrombectomy, IA	12 (3.0%)
Post-stroke medication	
Anti-platelets	338 (84.1%)
Anticoagulants	81 (20.1%)
Neuropsychologic test	
MoCA	16.0 [9.0–22.0]
Family caregiving status	0.0 [0.0–4.0]

Data are expressed as median [interquartile range] or number (percentage). TOAST, Trial of Org 10,172 in Acute Stroke Treatment; WBC, white blood cell; eGFR, estimated glomerular filtration rate; IV, intravenous; IA, intra-arterial; MoCA, Montreal Cognitive Assessment.

**Table 2:** Association between mRS scores and family caregiving status analyzed using the adjusted Linear Mixed Models.

	β	(95%CI)	*p*
Family caregiving status	0.17	(0.11–0.23)	<0.001
Time (in days) from discharge to 3 months after stroke onset	−0.01	(−0.01–0.00)	<0.001
Interaction	0.00	(0.00–0.00)	0.074

Lower score of family caregiving status represents less family burden and a better support environment in the home. 95%CI, 95% confidence interval.

## Data Availability

The raw data supporting the conclusions of this article will be made available by the authors upon request.
